# Cochlear Implant and Hearing Aid: Objective Measures of Binaural Benefit

**DOI:** 10.3389/fnins.2020.586119

**Published:** 2020-12-14

**Authors:** Tobias Balkenhol, Elisabeth Wallhäusser-Franke, Nicole Rotter, Jérôme J. Servais

**Affiliations:** Department of Otorhinolaryngology Head and Neck Surgery, Medical Faculty Mannheim, University Medical Center Mannheim, Heidelberg University, Mannheim, Germany

**Keywords:** cochlear implant, hearing aid, electroencephalography, auditory evoked potentials, source localization, speech recognition, bimodal benefit, auditory rehabilitation

## Abstract

Cochlear implants (CI) improve hearing for the severely hearing impaired. With an extension of implantation candidacy, today many CI listeners use a hearing aid on their contralateral ear, referred to as bimodal listening. It is uncertain, however, whether the brains of bimodal listeners can combine the electrical and acoustical sound information and how much CI experience is needed to achieve an improved performance with bimodal listening. Patients with bilateral sensorineural hearing loss undergoing implant surgery were tested in their ability to understand speech in quiet and in noise, before and again 3 and 6 months after provision of a CI. Results of these bimodal listeners were compared to age-matched, normal hearing controls (NH). The benefit of adding a contralateral hearing aid was calculated in terms of head shadow, binaural summation, binaural squelch, and spatial release from masking from the results of a sentence recognition test. Beyond that, bimodal benefit was estimated from the difference in amplitudes and latencies of the N1, P2, and N2 potentials of the brains’ auditory evoked response (AEP) toward speech. Data of fifteen participants contributed to the results. CI provision resulted in significant improvement of speech recognition with the CI ear, and in taking advantage of the head shadow effect for understanding speech in noise. Some amount of binaural processing was suggested by a positive binaural summation effect 6 month post-implantation that correlated significantly with symmetry of pure tone thresholds. Moreover, a significant negative correlation existed between binaural summation and latency of the P2 potential. With CI experience, morphology of the N1 and P2 potentials in the AEP response approximated that of NH, whereas, N2 remained different. Significant AEP differences between monaural and binaural processing were shown for NH and for bimodal listeners 6 month post-implantation. Although the grand-averaged difference in N1 amplitude between monaural and binaural listening was similar for NH and the bimodal group, source localization showed group-dependent differences in auditory and speech-relevant cortex, suggesting different processing in the bimodal listeners.

## Introduction

Cochlear implants (CI) are hearing prostheses that bypass defective sensory hair cells in the cochlea, allowing individuals with severe to profound sensorineural hearing loss to regain much of their hearing. As CI technology and surgical approaches have advanced, many patients with residual hearing in their opposite ear qualify for implantation. Thus today the bimodal group with electrically aided hearing in one ear and acoustically-aided hearing in the opposite ear represents the largest group of CI users (Holder et al., 2018). Beyond the fact that the better ear may change depending on the position of target and noise sources, and that bimodal fitting allows use of the ear that is best in any given situation, bimodal listening is expected to provide additional binaural benefits.

Binaural benefits are especially noticeable in challenging acoustic conditions, for instance when speech recognition is impeded by the presence of background noise. Normal hearing listeners (NH) are known to benefit from several binaural effects which have been well quantified in audiometric tests. These include: head shadow (HS), binaural summation (SU), binaural squelch (SQ), and spatial release from masking (SRM) (Bronkhorst and Plomp, 1988, 1989, 1990). Behavioral studies have investigated the amount of binaural benefit that exists in bimodal listeners, but results appear to be controversial (Schafer et al., 2011) and outcomes even include binaural interference, or worsening in comparison to hearing with the CI alone (Illg et al., 2014; Reiss et al., 2016). This may apply all the more so, since many CI listeners use hearing aids (HA) that are unsynchronized with and sometimes fitted independently of the CI. Thus, it is uncertain whether bimodal listeners benefit from a contralateral HA and which factors, either patient-based or provision-based, promote these benefits. In the current study, the CI was seen as the major channel for speech recognition, and we intended to explore whether addition of a HA posed a benefit. Therefore, all binaural benefits were calculated relative to monaural listening with the CI.

Audiometric binaural benefits have been investigated extensively in NH (Bronkhorst and Plomp, 1988, 1989, 1990), whereas objective measures are less well established, but should show as a difference in brain activity for conditions where a binaural effect on speech recognition is known to exist. Multichannel electrical recording (EEG) of auditory evoked potentials (AEP) can capture brain activity non-invasively. This method is compatible with CI use and time-sensitive enough to follow the rapid processing of speech signals (Balkenhol et al., 2020). Hence, responses evoked by monaural stimulus presentation can be directly compared to binaural presentation. Furthermore, comparing AEP traces and behavioral binaural effects for NH listeners, and potential discrepancies for bimodal listeners, may shed light on similarities as well as differences and on their behavioral relevance.

First aim of the current study was to describe AEP traces collected during monaural electrical and bimodal listening and to explore potential differences. In this context, AEP derived from NH listeners served as a template with which to compare the brain’s response in bimodal listeners. Some studies have investigated the effects of monaural vs. binaural presentation of auditory stimuli for NH (Henkin et al., 2015; Papesh et al., 2015), and one group performed initial studies on pure tone reception for bimodal listeners (Sasaki et al., 2009). In the current study monosyllable words and their time-reversed acoustic traces were presented monaurally and binaurally within speech-shaped noise. A spatial signal-to-noise constellation, which is known to be associated with a brain-mediated binaural benefit, but at the same time is practicable with monaural CI listening was used. Speech was delivered from the front (S0) and the noise source faced the HA ear (NHA).

Secondly, a related question was to explore whether brain plasticity in the course of adaptation to bimodal hearing plays a role. Obtaining a binaural benefit in the spatial S0NHA constellation requires combining the information from both ears in the central auditory system (Schafer et al., 2011). Therefore changes in the AEP are expected during acclimatization to bimodal hearing. This should be evidenced by a change in the differences between monaural and binaural responses recorded shortly after switch-on of the CI compared to those recorded after an extended time of CI experience. As variability is large in the CI group, this comparison requires repeated measurements for the same subjects. We previously showed that the obligatory N1 and P2 deflections of the brain’s AEP response approximated those of NH listeners within the first months of CI experience for binaural presentations, whereas the later event-related N2 potential did not show this effect (Balkenhol et al., 2020). Here we want to explore, whether differences exist between monaural and binaural responses, and whether these differences change with CI experience in the bimodal listeners.

Third aim of the study was to explore, whether monaural vs. binaural differences in the AEP correlate with binaural benefits evidenced by speech audiometry. If significant correlations exist, they could inform about aspects of the AEP response that may serve as an objective measure for binaural processing in bimodal listeners.

Taken together the present study explores whether bimodal listeners experience the same benefit that NH listeners experience, whether this needs time to develop, and whether potential differences in the AEP between monaural and binaural listening correlate with differences in behavioral performance.

## Materials and Methods

### CI Participants

The study protocol was approved by the Institutional Review Board of the Medical Faculty of Mannheim at Heidelberg University (approval no. 2014-527N-MA). Prior to inclusion, each participant provided written consent for participation in the study, and in accordance with the Declaration of Helsinki. All participants were compensated for their visits.

Other aspects of the influence of CI experience in this group of CI users were described earlier (Servais et al., 2017; Wallhäusser-Franke et al., 2018; Balkenhol et al., 2020). Whereas previous reports focused on tinnitus (Servais et al., 2017), subjective perception of the improvement in auditory abilities (Wallhäusser-Franke et al., 2018), and the development of bimodal hearing (Balkenhol et al., 2020), the current report focusses on the difference between monaural hearing with the CI and bimodal hearing.

Between 2014 and 2017, study participants were recruited from the patients of the CI Center at the University Medical Center Mannheim. Inclusion criteria comprised first-time unilateral CI provision, a HiRes 90K implant as chosen by the patient, continued HA use for the other ear, aged between 18 and 90 years, and speaking German as mother tongue. All patients who fulfilled these criteria were approached for inclusion. Exclusion criteria were assessed during an initial interview (T1) and included: more than mild cognitive deficit, as assessed by the DemTect Test (Kalbe et al., 2004), and presence of an internal stimulator apart from the CI. The initial interview, study inclusion (T1), and pre-surgery examination (T2) took place on the same day, usually the day before surgery. Patients received a CI on their poorer ear, while HA use was continued on the other ear. The CI was switched on 2–3 weeks following implantation. Post-implantation assessments T3 and T4 were scheduled for 3 and 6 months post-implantation, respectively. At each assessment, study participants went through audiometric tests, filled out standardized questionnaires, and underwent EEG recordings.

Twenty-seven patients with hearing loss for both ears, who planned to undergo unilateral CI provision were screened. One was excluded because of an exclusion criterion, while 26 were included in the study. Reasons for premature termination of the study were implantation of the contralateral ear (2 subjects), presence of an exclusion criterion that had not been disclosed at inclusion (1 subject), too much effort (1 subject), or reasons were not disclosed (2 subjects). Data of another 5 participants were excluded because of left-handedness (1 subject), missing AEP data (1 subject), or because of significant worsening of the HA ear during the study (3 subjects). This resulted in 15 participants who contributed data toward the AEP analysis. For demographic details of this group see Table 1. All study participants were right-handed native German speakers and used the NAIDA Q70 speech processor. Prior to implantation, 80% used a HA on both ears (Table 1), whereas post-implantation all non-implanted ears were aided by auditory amplification.

**TABLE 1:** Participant characteristics.

	CI group (*N*_CI_ = 15)	NH group (*N*_NH_ = 14)
**Age** Mean ± SD (range) in years	57.67 ± 14.95 (27–78)	57.21 ± 13.69 (24–76)
**Sex** female/male	12/3	12/2
**CI ear** left/right	8/7	10/4
**Lifetime with hearing impairment** Mean ± SD in %	CI ear: 53.72 ± 39.01 HA ear: 24.21 ± 19.01	
**HA use at future CI ear** yes/no	12/3	

#### History of Hearing Loss

At inclusion, all CI participants could communicate verbally when using their HA. Six participants reported hearing problems since early childhood, while 9 had post-lingual onset of profound hearing impairment. On average, severe hearing impairment of the CI ear existed for half of the participants’ lifetime, while the HA ear had a shorter duration of hearing loss (Table 1). Etiology was unknown for 73%, was due to sudden hearing loss in 2 cases, and one case each of Meniere’s disease and Stickler Syndrome.

### Normal Hearing Control Group

For each participant who completed the AEP measurement, a right-handed, age-, and sex-matched control with normal hearing was recruited. Control participants (NH) were recruited by word of mouth and from the employees of the University Medical Center Mannheim. Inclusion criteria were: German as native language, no past or present neurological, psychological or hearing problems and right handedness. NH underwent the same screening and undertook the same tests as the CI group. Data from one NH participant was not included because of poor AEP recording. Demographics for the 14 NH are presented in Table 1. Average hearing thresholds between 0.25 and 10 kHz for both ears of the 14 NH controls were 17.93 ± 10.32 dB.

### Setup for Speech Audiometry and EEG Recordings

Experimental setup is described in detail in Balkenhol et al. (2020). Speech comprehension tests and EEG recordings were performed in a dimly lit sound booth shielded against electromagnetic interference (IAC Acoustics, North Aurora, IL, United States). Participants sat in a comfortable armchair and were observed via glass window and camera.

Speech stimuli were presented in soundfield from a loudspeaker (M-Audio Fast Track Ultra USB Audio Interface and a BX5 near field monitor loudspeaker by inMusic Brand, Cumberland, RI, United States) 1 meter in front of the participant (0° azimuth: S0). Noise came from the same loudspeaker or from one of the two loudspeakers (same brand) at ±90° azimuth. Sound pressure level was always calibrated before testing and with ±0.5 dB accuracy (Brüel & Kjær 2250 sound level meter, Naerum, Denmark) (Letowski and Champlin, 2014).

#### Speech Audiometry

Speech audiometry was performed as in Balkenhol et al. (2020). An overview on tests and listening conditions is given in Table 2. Tests were performed for the following monaural and binaural listening conditions: CI alone (monCI), HA alone (monHA), CI and HA in combination (binaural/bimodal). For all monCI, the HA was removed and the ear was masked with white noise at 65 dB SPL through an insert earphone (AKG K350; Harman International, Stamford, CT, United States; earplug: Grason-Stadler Inc., Eden Prairie, MN, United States). For monaural listening with the HA, the CI was removed. In NH the contralateral ear was masked in both monaural listening conditions in the same way as for monCI.

**TABLE 2:** Experimental conditions.

	Test condition	Spatial arrangement	Test	Listening condition			HA ear muted for monCI
				T2	T3	T4	
	Quiet	S0	FBE	binaural (bin)	monCI, monHA, bimodal (bin)	monCI, monHA, bimodal (bin)	With white noise of 65 dB
		S0	OlSa				
Speech audiometry	OlSa noise	S0N0 S0NCI S0NHA	OlSa OlSa OlSa	binaural (bin)	monCI, monHA, bimodal (bin)	monCI, monHA, bimodal (bin)	With white noise of 65 dB

EEG	OlSa noise	S0NHA	30% monosyllable words, 70% time-reversed sound trace of monosyllable words	monCI (future CI ear), binaural (bin)	monCI, bimodal (bin)	monCI, bimodal (bin)	No

In all tests, speech was presented from the front (S0) by a male talker. Speech recognition in quiet was tested with the standard clinical German monosyllable test at 70 dB SPL (Freiburger Monosyllable Test or FBE: Hahlbrock, 1970; Löhler et al., 2014), and the adaptive version of a sentence test (Oldenburg matrix sentence test or OlSa: Wagener et al., 1999a,b, c). Speech recognition in speech-modulated noise (OlSa noise) was tested with the OlSa with noise delivered from the front (N0), from the speaker facing the CI (NCI), or the HA (NHA). While noise was constant at 60 dB SPL, speech level was changed adaptively starting from +10 dB SNR (signal to noise ratio). Listeners verbally repeated the word (FBE), or each word in a sentence (OlSa) as understood, and the experimenter entered the correct words. No feedback was given, lists were not repeated within sessions. FBE results comprised two lists of 20 words per listening condition with higher percentage indicating better speech recognition. For each test condition, twenty OlSa sentences were presented with the average calculated from the last ten sentences for 50% speech recognition in quiet (dB SRT) or the SNR needed for 50% correct comprehension in noise (dB SNR). Sequence of tests and lists was constant between participants and assessments but listening conditions were varied at random. While in the FBE higher values indicate better speech recognition, lower values in the OlSa are indicative of better speech recognition. Because monaural speech tests were not possible before implantation monaural vs. binaural comparisons are available only for the post-implantation assessments T3 and T4, and for NH.

#### Ear Dominance and Bimodal Benefit

The better ear, post-operatively, was determined for each speech test by subtracting the values obtained with monHA from the respective values with monCI. For a difference of more than 10% in the FBE, or 3 dB in the OlSa tests, aided hearing was defined as asymmetric and the better ear was determined. The 10% boundary for the FBE was chosen according to Müller-Deile (2009), the 3 dB boundaries for OlSa tests were derived from work by Litovsky et al. (2006).

Binaural benefits were calculated from OlSa tests as head shadow (HS), binaural summation (SU), binaural squelch (SQ), and spatial release from masking (SRM). All benefits were calculated relative to monaural listening with the CI ear. Calculations were carried out in such a way that binaural benefits will produce a positive value while binaural interference, i.e., worsening in the binaural condition, has a negative leading sign. Because lower values represent better speech recognition in OlSa tests, calculations derived from OlSa results were inverted. Calculations for NH were performed alike for monaural vs. binaural listening.

The binaural benefits HS, SU, and SQ were calculated from OlSa results according to Schleich et al. (2004). HS was calculated as follows:

HS_monCI_ = S0NCI_monCI_ – S0NHA_monCI_.                                                                                                                                (1)

Binaural loudness summation (SU) was calculated for speech presented in quiet (SU_Q_) and for speech presented with noise from the same source (SU_N_) in the following way:

SU_Q_ = S0_b__in_ – S0_m__onCI_,                                                                                                                                                              (2)

SU_N_ = S0N0_b__in_ – S0N0_m__onCI_.                                                                                                                                                  (3)

Binaural SQ was calculated for the condition with lateral noise contralateral to the monaurally active ear (Schleich et al., 2004), here the CI ear:

SQ = S0NHA_bin_ – S0NHA_monCI_.                                                                                                                                              (4)

This spatial signal to noise constellation is the same as the one used during EEG recordings (see section “EEG Recordings”). A measure of SRM was derived by subtracting speech recognition within lateral noise (S0NHA) from the condition of collocated speech and noise (S0N0) for monaural listening with the CI ear:

SRM_monCI_ = S0N0_m__onCI_ – S0NHA_monCI_                                                                                                                              (5)

and for binaural listening:

SRM_bin_ = S0N0_b__in_ – S0NHA_bin_.                                                                                                                                             (6)

Normal distributions of auditory outcomes were checked with the Shapiro-Wilk test (Shapiro and Wilk, 1965) and by inspection of outcome distributions. Monaural vs. binaural comparisons for speech tests in quiet (FBE, OlSa S0) were tested for significance by planned comparisons with parametric (*t* values) or non-parametric tests (*z* values) depending on normality. Statistical significance of differences for speech recognition in noise were determined for T3 and T4 assessments, and for NH with 3 spatial conditions (S0N0, S0NCI, S0NHA) × 2 listening conditions (monaural, binaural) with repeated-measures ANOVAs by MATLAB’s Statistics and Machine Learning Toolbox (R2018a) (Mathworks, Natick, MA, United States). Because of small sample size, Greenhouse-Geisser adjustment was used to correct against violations of sphericity. Given that a significant main effect existed, *post hoc* two-tailed paired samples *t* tests were performed and corrected for multiple comparisons according to Tukey-Kramer. Whether bimodal HS, SU, SQ, and SRM effects differed significantly from zero was determined with one-sample *t* tests. To correct for multiple testing, Bonferroni-corrected significance limens equivalent to the *p* value that indicates a trend (^+^*p* < 0.1), a significant difference (^∗^*p* < 0.05), or a highly significant difference (^∗∗^*p* < 0.01) are given together with the uncorrected *p* value. Differences in HS, SU, SQ, and SRM between T3, T4, to NH were tested for significance with Dunnett’s multiple comparison test (Dunnett, 1955; Dunlap et al., 1981). Group means (Mean) together with their standard deviations (SD) are used throughout the text if not indicated otherwise. Correlation analyses for audiometric measures were performed with SPSS25 (SPSS/IBM, Chicago, IL, United States).

### EEG Recordings

As described in Balkenhol et al. (2020) EEG was continuously recorded from 62 active Ag/AgCl surface electrodes arranged in an elastic cap (g.LADYbird/g.GAMMAcap; g.tec Medical Engineering GmbH, Austria) according to the 10/10 system (Oostenveld and Praamstra, 2011), Fpz served as ground. Two active Ag/AgCl electrodes (g.GAMMAearclip; g.tec) were clipped to the earlobes. The electrooculogram (EOG) was monitored with 4 passive Ag/AgCl electrodes (Natus Europe GmbH, Germany) placed below and at the outer canthi of the eyes. Electrodes located above or close to CI or HA were not filled with gel [Mean ± SD (range): CI: 3 ± 1.1 (1–5); HA: 1 ± 0.5 (0–2)] and were interpolated during post-processing. Impedances were below 5 kOhm for passive electrodes, and below 30 kOhm for active electrodes. Sampling frequency was 512 Hz with 24-bit resolution (biosignal amplifier: g.HIamp; g.tec). Data acquisition and playback of the stimuli were controlled by MATLAB/Simulink R2010a (Mathworks, Natick, MA, United States) with custom MATLAB scripts. Real-time access to the soundcard was realized with the playrec toolbox^1^. A trigger box (g.TRIGbox; g.tec) was used to mark stimulus onsets and offsets and to record push button activity (see section “Task and Procedure”).

#### Stimuli

Stimuli were German monosyllables from the FBE spoken by a male talker (Hahlbrock, 1970). Reversals were generated by time-reversing the audio tracks of these monosyllables. Only reversals that did not resemble a German word as judged by the lab members were used. In total, 269 words and 216 reversals, with a mean duration of 770 ± 98 ms (484–1,035 ms) were used. Lists were generated randomly from the complete set with 75 stimuli in a stimulation block. 30% of these stimuli were words and 70% were reversals. Lists were not repeated during an assessment. During all stimulation blocks OlSa noise at 60 dB SPL (Wagener et al., 1999a,b, c) was delivered toward the HA ear or the ear that was not active in the monaural condition in NH controls (azimuth ±90°: NHA).

#### Task and Procedure

Participants were instructed to face the loudspeaker in front where the signals originated (S0), to close their eyes, and not to move during recording. Their task was to respond to the infrequent words by pressing a button after hearing a signal sound (white noise, 75 dB SPL, 50 ms) that followed 1,000 ms after offset of each word and reversal. The button press served both to maintain alertness and to calculate the percentage of words identified within a stimulation block which was used to calculate binaural squelch (SQ_AEP_) for this condition (for calculation see section “Bimodal Benefits”). Inter-stimulus intervals between the end of the signal sound and the start of the next stimulus were 1,900 ± 200 ms resulting in 75 stimuli per 5 minutes presentation block. During the entire presentation block continuous OlSa noise was played from the loudspeaker facing the HA ear (NHA). Each block was followed by a break during which participants could relax. All participants received the same randomized stimulus sequence within each block, whereas the sequence of monaural and binaural listening conditions varied. Overall, 297 ± 55 responses were recorded for monaural and 303 ± 61 for binaural listening conditions.

To avoid ceiling and floor effects, signal to noise ratio was individually set to achieve 70% correct detection of words (dB SNR) as ascertained in practice runs prior to recording. If rates deviated substantially from this criterion, the procedure was repeated with an adjusted presentation level. If button press occurred before the signal sound, that AEP was excluded from analysis. At T4, two familiarization blocks were performed using the same SNR as at T3.

#### EEG Pre-processing

EEG data were pre-processed offline with MATLAB R2018a (Mathworks, Natick, MA, United States) with the EEGLAB toolbox (version 13.3.2b) (Delorme and Makeig, 2004), and custom MATLAB scripts as described in Balkenhol et al. (2020). Raw data were: (1) re-referenced to linked earlobes, (2) low-pass filtered with 64 Hz cut-off and (3) high-pass filtered with 0.5 Hz cut-off using finite impulse response (FIR) filters, and (4) segmented into epochs from −300 to 2,200 ms relative to stimulus onset. Epochs with amplitudes exceeding ±150 μV in single channels or with non-stereotyped artifacts, classified by kurtosis and joint probability (threshold: 3 SD), were highlighted during visual inspection. Final rejection of epochs and the identification of poor electrode channels [CI group Mean ± SD (range): 0.8 ± 1.5 (0–7); NH group Mean ± SD (range): 0.9 ± 1.1 (0–3)] were performed by experienced lab members.

Next, EOG artifacts were removed automatically using a second-order blind identification (SOBI) and independent component analysis (ICA) (Molgedey and Schuster, 1994; Onton et al., 2006; Delorme et al., 2007), as described in Balkenhol et al. (2013).

The CI induced narrow- and wide-band EEG components above 25 Hz in response to words and reversals. These were removed with SOBI ICA using an automated artifact removal algorithm developed for this study which identifies artifacts in the independent components based on power distribution. While narrow-band artifacts were automatically detected by a spectral peak search algorithm, wide-band artifacts were identified by their average power in the high frequencies (40–256 Hz), relative to power in low frequencies (3–25 Hz). Components were removed if spectral power in the high-frequency interval exceeded power in the low-frequency interval (Balkenhol et al., 2020).

Then, muscle artifacts, heartbeat activity, and other sources of non-cerebral activity were visually identified on independent component scalp maps and their power spectra (Luck, 2014), and removed by back-projecting all but these components. Finally, unfilled and channels of poor quality were interpolated by spherical splines. On average, 14% of the AEP were removed while 256 ± 54 responses remained per participant and assessment.

#### EEG Data Analysis

Amplitudes and latencies were computed for the N1, P2, and N2 deflections for monaural and binaural listening conditions and for each stimulus category. Binaural-monaural differences were calculated.

As described in Balkenhol et al. (2020) data analysis was performed in MATLAB R2018a (Mathworks, Natick, MA, United States) with the fieldtrip toolbox (version 20170925^2^; Oostenveld et al., 2011) and custom MATLAB scripts. Computations are based on subject averages across all 62 electrodes, and separately for the categories “words” (all responses to word stimuli) and “reversals” (all responses to reversed stimuli). For baseline correction, the pre-stimulus mean from –150 to –50 ms was subtracted from each epoch. Differences in intensity rise times between stimuli were corrected by delaying the onset trigger to the first time point when a stimulus reached 50% of its maximal amplitude. Amplitudes were calculated for the time intervals from 80–180 ms (N1), 180–330 ms (P2), and 370–570 ms (N2) (Luck, 2014). N1, P2, and N2 latencies were quantified by the 50% area latency measure according to Liesefeld (2018) and as described in Balkenhol et al. (2020). In short, peak-to-peak amplitude distance to the preceding peak was determined, the baseline which divided amplitudes in half was identified, and the time point that splits this area in half was calculated.

Statistical analysis was performed with MATLAB’s Statistics and Machine Learning Toolbox (R2018a) and custom scripts. Depending on distribution of the data, parametric or non-parametric tests were used. Amplitudes and area latencies for N1, P2, and N2 responses corresponding to “words” and “reversals” were subjected to separate Dunnett’s multiple comparison procedures to compare CI group results at T2, T3, and T4 with the NH group for monCI and bimodal listening conditions (Dunnett, 1955; Dunlap et al., 1981). For comparisons with significant main effects, *post hoc t* or Wilcoxon tests were performed. A value of *p* < 0.05 was considered to be statistically significant, while *p* < 0.1 indicated a trend.

#### Source Localization

Source localization analysis for the N1 interval was performed with MATLAB’s fieldtrip toolbox and time-domain based eLORETA (Pascual-Marqui, 2007, 2009) using the “colin27” head model (Holmes et al., 1998). Monte-Carlo estimates of probability were derived by non-parametric randomization tests (*N*_r_ = 1,000, two-sided). Leadfield resolution was 5 mm, statistical analysis was performed on dipole power, and a false discovery rate (FDR) was used to correct for multiple comparisons. A detailed description of this procedure is given in Balkenhol et al. (2020).

## Results for Speech Audiometry

For ease of reporting and interpretation, differences calculated from OlSa tests have been inverted such that binaural benefits will be reported with positive numbers while binaural interference is indicated through negative numbers. This is despite the fact that a lower score represents better performance for the OlSa tests.

### Development of Speech Recognition in the CI-Aided Ear

**FIGURE 1:**
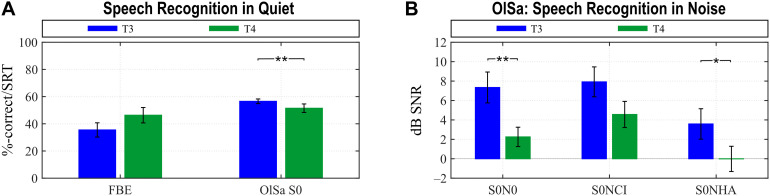
Improvement of hearing with the CI ear between T3 and T4. Significant improvements are seen for various test constellations in quiet **(A)** and background noise **(B)**. In the FBE higher values (%-correct) indicate better performance, whereas in all OlSa tests, lower values indicate better performance. Group means with their standard errors are shown (**p < 0.002, *p < 0.01).

Average scores are presented in Figure 1 for speech recognition in quiet (FBE, OlSa S0), and with background noise from different directions (OlSa: S0N0, S0NCI, S0NHA). For the CI group, monaural speech comprehension tests were not performed pre-implantation due to the inability of many of the participants to complete these tests. In addition, one participant was not able to complete some OlSa tests with monaural CI-aided listening at T3 and T4.

Post-implantation, paired *t* tests (*t* values) and Wilcoxon tests in case of non-normality (*z* values) showed significant improvements of speech recognition with the CI ear between T3 and T4 for the OlSa S0 (*z* = 3.296, ^∗∗^*p* < 0.001), S0N0 (*t* = 3.300, ^∗∗^*p* < 0.001), and S0NHA (*z* = 2.638, ^∗^*p* < 0.009) conditions when applying the Bonferroni-corrected significance limen for significant ^∗^ (*p* < 0.01) and highly significant ^∗∗^ (*p* < 0.002) differences (Figure 1).

### Ear Dominance

At the onset of the study, the CI ear was expected to become the better ear post-implantation. The study population included individuals with substantial amounts of aidable hearing on the HA side, however. So, at T4, hearing abilities were equally distributed across ears, with about one third each of the participants falling into the “symmetric”, “better CI ear”, or “better HA ear” categories according to average performance on all speech perception tests (Table 3). In contrast, at T3, the HA ear was the better ear for more than half of the participants, while about 30% had symmetric speech recognition, and the CI ear was the better ear for 16%. This distribution differed considerably between test conditions as can be seen in Table 3.

**TABLE 3:** Better ear during speech recognition.

Speech recognition test	T3			T4			NH		
	Symmetric *N*_S,T__3_ (%)	CI ear better *N*_CI,T__3_ (%)	HA ear better *N*_HA,T__3_ (%)	Symmetric *N*_S,T__4_ (%)	CI ear better *N*_CI,T__4_ (%)	HA ear better *N*_HA,T__4_ (%)	Symmetric *N*_S,NH_ (%)	Designated CI ear better *N*_CI,NH_ (%)	Designated HA ear better *N*_HA,NH_ (%)
FBE	5 (33.3)	2 (13.3)	8 (53.3)	2 (13.3)	5 (33.3)	8 (53.3)	14 (100)	0	0
OlSa S0	3 (20.0)	4 (26.7)	8 (53.3)	1 (6.7)	7 (46.7)	7 (46.7)	7 (50)	4 (28.6)	3 (21.4)
OlSa S0N0	6 (40.0)	2 (13.3)	7 (46.7)	8 (53.3)	5 (33.3)	2 (13.3)	14 (100)	0	0
OlSa S0N_ipsi_	5 (33.3)	2 (13.3)	8 (53.3)	6 (40.0)	4 (26.7)	5 (33.3)	9 (64.3)	1 (7.1)	4 (28.6)
OlSa S0N_contra_	4 (26.7)	2 (13.3)	9 (60.0)	8 (53.3)	3 (20.0)	4 (26.7)	9 (64.3)	2 (14.3)	3 (21.4)
Mean	4.6 (30.7)	2.4 (16.0)	8 (53.3)	5 (33.3)	4.8 (32.0)	5.2 (34.7)	10.6 (75.7)	1.4 (10.0)	2 (14.3)

For comparison, about 76% of the NH listeners showed symmetric performance. Symmetry was not perfect, however, and mainly pertained to FBE and OlSa S0N0 (Table 3). As a right ear advantage has been reported for speech perception (Westerhausen et al., 2015), data of NH were additionally screened for right vs. left ear comparisons. Since there were no significant differences for speech recognition achieved with either ear for any of the conditions tested in the current NH group, this issue was not pursued further.

The S0NHA constellation in the OlSa test was closest to the spatial distribution of speech and noise sources during EEG recordings. For this condition audiometric outcomes imply addition of a better HA ear in the bimodal listening condition for 60% of CI participants at T3, and addition of an equal ear for 53.3% at T4 which was closer to the situation in NH where symmetric speech recognition was found for 64.3% (Table 3).

### Monaural vs. Binaural Comparisons

#### NH Group

**FIGURE 2:**
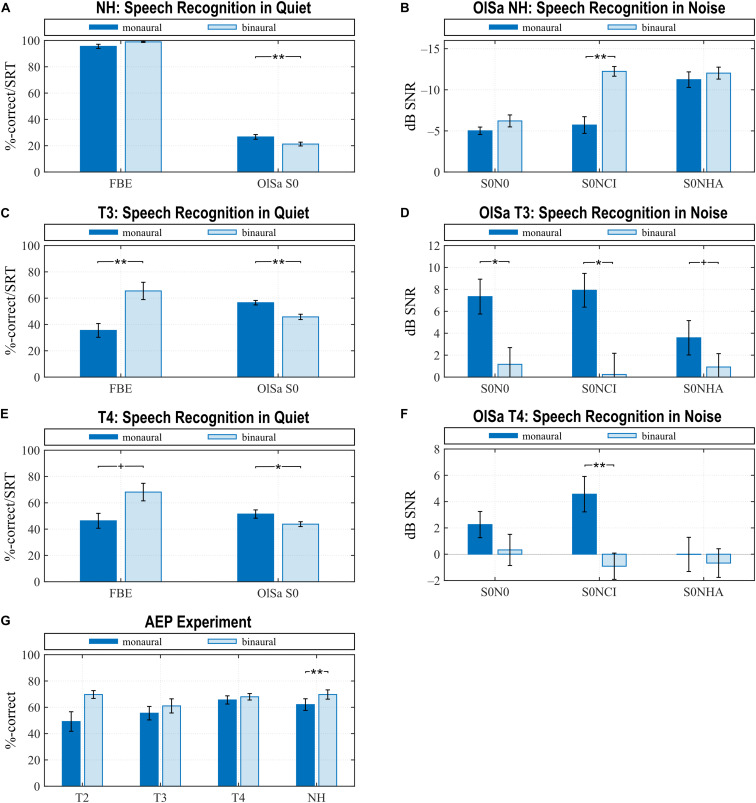
Speech perception with FBE and OlSa tests **(A–F)** and assessed in the AEP experiment **(G)**. Higher values signal better speech recognition in the FBE and AEP conditions, whereas lower values indicate better speech recognition in OlSa tests. Note the reversed vertical scale in **(B)**. Perception is best in NH listeners **(A,B,G)**, worst shortly after CI provision **(C,D,G)** and improves with CI experience **(E–G)**. Statistically significant differences between monaural listening with the CI or the designated CI ear in NH and binaural speech recognition was observed for several test conditions. Due to insufficient monaural hearing, monaural data at T2 are only available for the AEP condition, where a significant difference existed between monaural and binaural listening. While behavioral results from the AEP experiment at T3 and T4 did not evidence a significant difference between listening conditions, behavioral tests showed significantly better bimodal speech recognition at T3 for all test conditions and at T4 for the S0NCI condition. Regarding the spatial arrangement of speech and noise sources, the AEP condition is closest to S0NHA. Means and their standard error are shown (**p < 0.01, *p < 0.05, trends ^+^*p* < 0.1).

NH listeners gained the largest binaural benefits, therefore results from this group are presented first, and results of the CI group are compared to them. Many NH performed the FBE with a ceiling effect (monCI: 95.5 ± 5.9%; binaural: 98.9 ± 1.6%), and the difference between listening conditions (*z* = –2.203, *p* = 0.028) failed the Bonferroni-corrected significance limen of *p* < 0.025. In contrast, recognition in the OlSa S0 test was significantly better for sentences presented binaurally (*t* = 4.806, *p* < 0.0004) (Figure 2A). A 3 × 2 (noise direction: S0N0, S0NCI, S0NHA × listening condition: monaural, binaural) repeated-measures ANOVA revealed a significant main effect for noise direction [*F*(2,26) = 61.258, *p* < 3⋅10^–10^] and listening condition [*F*(1,13) = 64.599, *p* < 3⋅10^–6^], with a significant interaction between these factors [*F*(2,26) = 24.916, *p* < 2⋅10^–5^]. *Post hoc* tests focused on listening condition and revealed significantly better results for binaural listening when noise was presented at the side of the monaurally active ear (S0NCI: *t* = 8.630, *p* < 2⋅10^–5^). The difference between monaural and binaural presentation remained insignificant for noise from the same source (S0N0: *t* = 1.925, *p* = 0.431), or for noise presented from the side of the monaurally inactive ear (S0NHA: *t* = 1.633, *p* = 0.593) (Figure 2B).

#### CI Group

At T3, the bimodal condition most often equaled addition of an equally or better performing HA ear (Table 3), and significant improvements for bimodal listening compared to monCI were evidenced for all speech comprehension tests. For tests in quiet, planned comparisons evidenced significant effects for FBE (*t* = –4.085, *p* < 0.002) and OlSa S0 (*z* = 3.296, *p* < 0.001), when applying the Bonferroni-corrected significance limen of *p* < 0.025 (Figure 2C). A 3 × 2 repeated-measures ANOVA for hearing in noise revealed a significant main effect for noise direction [*F*(2,26) = 69.560, *p* < 0.009] and listening condition [*F*(1,13) = 14.538, *p* < 0.003], as well as a significant interaction [*F*(2,26) = 4.421, *p* < 0.041]. *Post hoc* tests for monCI vs. bimodal hearing confirmed significant improvements with bimodal hearing for S0N0 (*t* = 3.385, *p* < 0.045) and S0NCI (*t* = 3.556, *p* < 0.033), whereas a trend was observed for S0NHA (*t* = 3.129, *p* = 0.069) (Figure 2D).

By the time of the T4 sessions, the distribution of performance between ears had changed (Table 3), and significant improvements between bimodal and monaural electric hearing existed, although not for all conditions (Figures 2E,F). In quiet, speech perception improved for bimodal hearing for OlSa S0 (*z* = 2.727, *p* < 0.007) but not for the FBE (*z* = –2.047, *p* = 0.041) when applying the Bonferroni-corrected significance limen of *p* < 0.025 (Figure 2E). Significant main effects for noise direction [*F*(2,26) = 5.999, *p* < 0.017] and listening condition [*F*(1,13) = 7.877, *p* < 0.015] were derived by a 3 × 2 repeated-measures ANOVA. The interaction effect was also statistically significant [*F*(2,26) = 10.374, *p* < 0.002]. *Post hoc* tests for monCI vs. bimodal showed significant improvements in the bimodal condition when noise was presented from the CI side (S0NCI: *t* = 5.445, *p* < 0.002), but no difference existed for S0N0 (*t* = 2.362, *p* = 0.282) or S0NHA (*t* = 0.669, *p* = 0.983) (Figure 2F).

### Binaural Benefits

Binaural benefits were calculated for the addition of the HA ear relative to monaural listening with the CI as head shadow (HS), binaural summation in quiet (SU_Q_) and noise (SU_N_), as binaural squelch (SQ, SQ_AEP_), and spatial release from masking (SRM) for monaural (SRM_monCI_) and binaural listening (SRM_bin_) (Table 4).

**TABLE 4:** Binaural Benefit when adding the HA ear. Shown are Mean ± SD (range).

Group	HS_monCI_ = S0NCI_monCI_ – S0NHA_monCI_ in dB SNR	SRM_monCI_ = S0N0_monCI_ – S0NHA_monCI_ in dB SNR	SRM_bin_ = S0N0_bin_ – S0NHA_bin_ in dB SNR	SU_Q_ = S0_bin_ – S0_monCI_ in –dB SRT	SU_N_ = S0N0_bin_ – S0N0_monCI_ in –dB SNR	SQ = S0NHA_bin_ – S0NHA_monCI_ in –dB SNR	SQ_AEP_ = S0NHA_bin,AEP_ – S0NHA_monCI,AEP_ in %-correct
CI T2	–	–	–	–	–	–	22.06 ± 33.37 (−14.1−86.2)
CI T3	4.34 ± 4.09 (−3.9−10.5)	3.76 ± 4.23 (−5.9−10.3)	0.25 ± 3.96 (−5.6−10.9)	10.26 ± 9.36 (0.7−33.4)	6.16 ± 6.81 (−4.1−17.8)	3.06 ± 3.67 (−3.2−9.7)	5.53 ± 17.43 (−18.5−35.9)
CI T4	4.58 ± 3.99 (−5.5−10.3)	2.44 ± 3.26 (−3.0−7.6)	1.00 ± 3.98 (−6.4−9.2)	7.68 ± 13.68 (−1.7−53.3)	1.93 ± 3.16 (−4.0−6.9)	0.96 ± 5.40 (−13.2−9.1)	2.39 ± 10.06 (−15−27)
NH	5.52 ± 2.69 (0−9.3)	6.22 ± 2.20 (1.1−9.3)	5.81 ± 3.27 (−2.9−9.7)	5.43 ± 4.23 (−1.1−16.8)	1.20 ± 2.33 (−1.4−8.4)	0.79 ± 1.82 (−3.0−2.7)	7.66 ± 5.80 (−3.3−19.6)

For NH, significant HS and SRM effects were estimated (HS: *t* = 7.670, *p* < 4⋅10^–6^; SRM_monCI_: *t* = 10.567, *p* < 10^–7^; SRM_bin_: *z* = 3.170, *p* < 0.002). SRM_monCI_ and SRM_bin_ did not differ (*z* = −0.699, *p* = 0.485). SU_Q_ was higher than SU_N_ (*z* = 3.107, *p* < 0.002), and while SU_Q_ was significantly different from zero (*z* = 3.323, *p* < 0.002), SU_N_ failed significance after Bonferroni correction (*z* = 2.198, *p* = 0.028). Also, SQ calculated from OlSa S0NHA remained insignificant (*t* = 1.633, *p* = 0.126), whereas SQ_AEP_ derived from the button-press response during EEG recordings attained significance (*t* = 4.935, *p* < 0.0003). The corrected significance limen for all tests against zero was *p* < 0.0071.

In the CI group, a significant HS of similar magnitude as in NH was present at both post-CI assessments (Dunnett’s test: *F* = 0.413, *p* = 0.665; T3 vs. NH: *p* = 0.600; T4 vs. NH: *p* = 0.719), and one-sample *t* tests against zero with a Bonferroni-corrected significance limen of *p* < 0.0071 evidenced its significance at T3 (*t* = 3.968, *p* < 0.002) and T4 (*t* = 4.290, *p* < 0.001).

A difference between NH and bimodal listeners existed regarding SRM_monCI_ and SRM_bin_ with CI listeners benefiting significantly less (Dunnett’s test: SRM_monCI_: *F* = 4.649, *p* < 0.016; SRM_bin_: *F* = 9.235, *p* < 0.0005). Whereas SRM_monCI_ was significantly different from zero at T3 (*t* = 3.327, *p* < 0.006), significance did not survive Bonferroni correction at T4 (*t* = 2.793, *p* = 0.015), and SRM_bin_ was far from reaching significance at both assessments (T3: *t* = 0.241, *p* = 0.813; T4: *t* = 0.973, *p* = 0.347). The large reduction between SRM_monCI_ and SRM_bin_ for the bimodal listeners, especially at T3 (Table 4) did not attain significance when tested against NH where SRM_monCI_ and SRM_bin_ were similar (Dunnett’s test: *F* = 1.430, *p* = 0.251).

Bimodal listeners benefited significantly from binaural summation at T3 (SU_Q_: *t* = 4.098, *p* < 0.002; SU_N_: *t* = 3.385, *p* < 0.005) but less so at T4 with SU_N_ losing significance when corrected for multiple comparisons (SU_Q_: *z* = 2.723, *p* < 0.007; SU_N_: *t* = 2.362, *p* = 0.033). Dunnett’s test comparing T3 and T4 assessments with NH yielded a significant main effect for SU_N_ (*F* = 4.971, *p* < 0.012; T3 vs. NH: *p* < 0.012; T4 vs. NH: *p* = 0.872), but not for SU_Q_ (*F* = 0.819, *p* = 0.448). Similar to NH, at T4, SU dropped considerably between quiet and noise (T4: *z* = 2.101, *p* < 0.036; NH: *z* = 3.107, *p* < 0.002).

As in NH, the benefit derived from binaural SQ in the OlSa test did not attain the Bonferroni-corrected significance limen of *p* < 0.0071 at either assessment (T3: *t* = 3.129, *p* < 0.008; T4: *t* = 0.669, *p* = 0.516). Therefore, no further calculations were performed with this measure. In contrast, SQ_AEP_, which attained significance in NH, was not different from zero pre- (T2: *z* = 2.273, *p* = 0.024) or post-implantation (T3: *t* = 1.229, *p* = 0.239; T4: *t* = 0.921; *p* = 0.373). Statistical comparisons between CI assessments and NH evidenced a significant main effect (Dunnett’s test: *F* = 2.792, *p* < 0.05), while *post hoc* comparisons showed no significant differences to NH which may be a consequence of differences in SQ_AEP_ between assessments and heterogeneity of the bimodal listeners (Table 4).

Taken together, monaural intelligibility with the CI ear improved with CI experience, while evidence for binaural processing was limited to a positive SU_Q_ given that HS is essentially a monaural effect.

### Correlations Between Audiometric Measures

Correlations were tested on an exploratory basis for NH and the T4 assessment and not corrected for multiple comparisons. Bivariate comparisons between the different binaural/bimodal effects, and between these effects and PTA-4 as well as with PTA-4 asymmetry were calculated. Only significant correlations are reported. Several significant positive as well as negative correlations were found (Table 5). Mostly, these were present for either the NH or the CI group, but not for both. The only exception was the significant inverse correlation between SU_Q_ and PTA-4 asymmetry, which in CI listeners represented the aided PTA-4 of the CI and HA ears. For both groups, the correlation had the same direction and magnitude. This indicated that, on one hand addition of a better HA ear produces a larger SU_Q,_ and that on the other hand, lower asymmetry between ears is associated with a larger SU_Q_ if the CI ear is an equal or the better ear.

**TABLE 5:** Correlation matrix between Binaural Benefits in the bimodal group at T4 and in NH controls.

Binaural Effect *r* and *p* value	SRM_monCI_		SU_Q_		SU_N_		SQ		SQ_AEP_		PTA-4 CI		PTA-4 HA		PTA-4 asymmetry	
	CI T4	NH	CI T4	NH	CI T4	NH	CI T4	NH	CI T4	NH	CI T4	NH	CI T4	NH	CI T4	NH
HS	0.819 <*0.0001*		–0.579 *0.030*													
SRM_monCI_			–0.653 *0.011*					–0.604 *0.022*		–0.619 *0.018*		–0.542 *0.045*				
SRM_bin_						–0.839 *<0.0001*										
SN_Q_							0.608 *0.021*					0.756 *0.001*			–0.698 *0.005*	–0.655 *0.011*
SU_N_							0.603 *0.023*									
SQ_AEP_														0.927 *<0.0001*		
PTA-4 CI													0.926 *<0.001*		–0.723 *0.003*	

*Pearson correlation coefficients r and corresponding p values (in italics) are shown.*

## Results for EEG Recordings

### AEP: Monaural vs. Binaural and Words vs. Reversals Comparisons

Results from NH listeners are described first and then compared to those of the CI group.

**FIGURE 3:**
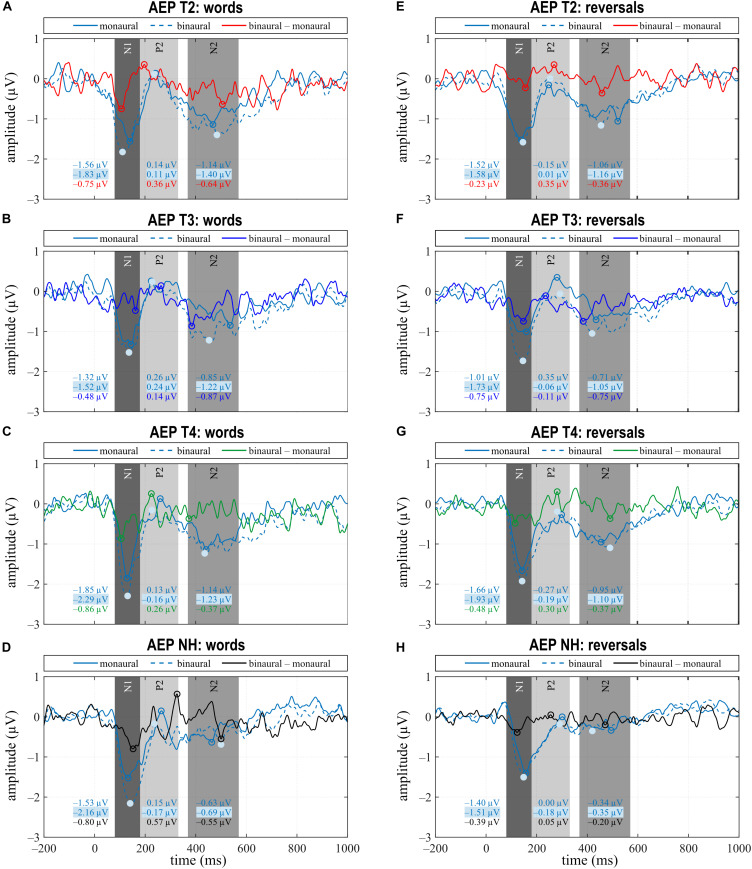
Grand averages for monaural, binaural listening conditions, and the difference binaural – monaural for the categories “words” **(A–D)** and “reversals” **(E–H)** of the CI (T2–T4) and NH group. **(A–H)** Time intervals with N1, P2, and N2 responses are shaded in different grays.

**FIGURE 4:**
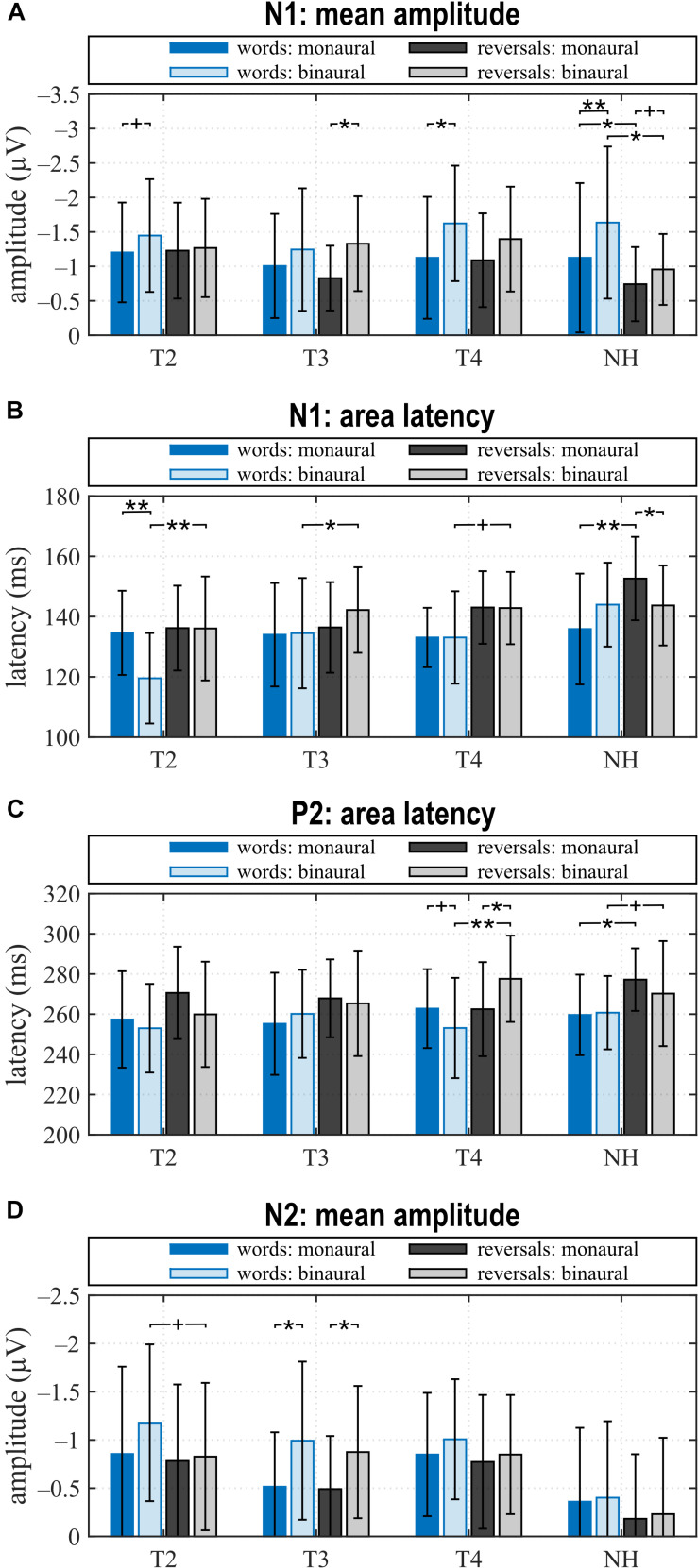
Quantitative AEP results: **(A)** mean amplitude and **(B)** area latency of the N1, **(C)** area latency of P2, and **(D)** mean amplitude of N2 for the categories “words”, “reversals”, and listening conditions monCI and binaural. **(A–D)** Means with their standard deviations are shown; significant differences between stimulus categories and listening conditions are indicated (**p < 0.001, *p < 0.05, and trends ^+^*p* < 0.1).

In NH, most conspicuous differences between listening conditions and stimulus categories pertained to N1 with N1 amplitudes differing significantly for all comparisons (Figures 3, 4). Only the monaural to binaural comparison following reversals failed full significance, but revealed a trend. N1 responses toward words were larger, and the most negative peak was seen following word presentation with binaural listening. A significant difference in N1 latency was observed between stimulus categories for monaural presentation with the response occurring earlier after words. In addition, a difference existed in response to reversals with the N1 response occurring significantly earlier with binaural listening. Whereas P2 amplitudes were similar for all conditions, P2 latencies differed between stimulus categories with shorter latencies following words. While this difference became significant for monaural listening, a trend toward significance was seen for the binaural response. N2 amplitude was low for all conditions.

Grand average of the CI group for N1 and P2 was comparable to NH, but deviated for N2 (Figures 3, 4). Pre-implantation, N1 amplitude did not differ between listening conditions or stimulus categories, while significant differences between listening conditions were observed post-implantation. Following words, N1 was larger with bimodal hearing. This difference extended with bimodal experience and became significant at T4 which paralleled the significant difference observed in NH in direction and magnitude. In contrast, the difference of N1 negativities between listening conditions following reversals peaked at T3 when this difference became statistically significant, and failed significance at T4. In contrast to NH, N1 amplitude did not differ between stimulus categories at either assessment, and N1 latencies to reversals were delayed relative to words in the binaural condition. This difference in delay was highly significant before implantation (T2), attained significance at T3, and reduced to a trend at T4. Thus, differences in the N1 response between listening conditions approximated those seen in NH within 6 months of CI experience, while absence of a difference between stimulus categories did not parallel the situation in NH.

Regarding P2, a significant difference in amplitude between monCI and bimodal listening following reversals was observed at T3, whereas further significant differences pertained to P2 latency at T4. At T4, significant latency differences existed between listening conditions, but in opposite directions for word and reversal stimulus categories. Whereas P2 latency following words was significantly shorter with bimodal listening, latency following reversals was significantly shorter for monCI. In addition, a highly significant difference existed between stimulus categories in the bimodal listening condition. Only the latter had a parallel in NH with a trend toward significance for the latency difference between stimulus categories with binaural listening and a later response to reversals.

While N2 was almost absent in NH, it was a prominent negative deflection in the CI group at all assessments and for all conditions. A trend toward a larger response to words than reversals existed at T2 for binaural listening, while significant differences between listening conditions were observed at T3 for both stimulus categories.

### Source Localization of Monaural/Binaural Differences

**FIGURE 5:**
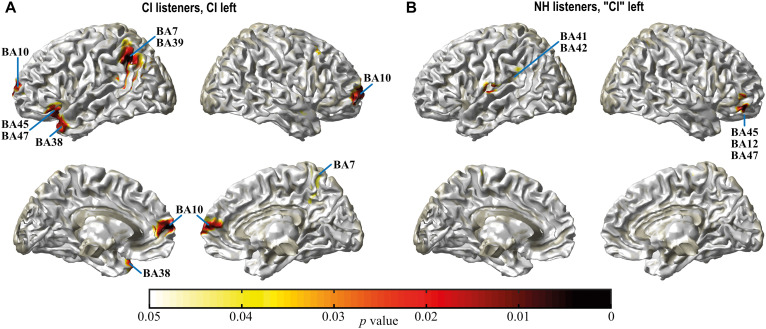
Spatial spread of monaural vs. binaural activation in CI listeners at T4 **(A)** and in NH **(B)** during the N1 interval. Only differences in regions bordering the surface or the midline of the cortex are visible in this illustration. For a complete list of areas with differential activation (see Table 6). Differences are more widespread in CI listeners compared to NH. Whereas in NH differential activation located to primary and secondary auditory cortex (BA41, 42), it pertained to auditory association cortex related to speech processing (BA21, 38, 39) and a region related with these areas (BA7) in the bimodal listeners. Darkening of the color scale indicates decreasing *p* values or higher significance.

Time domain eLORETA analyses were computed for the N1 response at T4 and in NH for monaural vs. binaural hearing. As for the majority of study participants the left ear was the ear that was stimulated in the monaural listening condition (Table 1), this analysis was performed for the 8 study participants with a CI on their left ear and the 10 NH with left ear monaural stimulation. Significant activation differences between monaural and binaural listening were observed (Figure 5), but locations differed between NH and CI groups. Table 6 lists brain structures with a significant difference between listening conditions in at least 20% of their voxels.

**TABLE 6:** Source localization results for subjects with CI on left ear.

		% significant (mean *t* values)	
Frontal lobe	Voxel in ROI	CI listeners	NH listeners
SFG, Superior Frontal Gyrus, medial area BA10	8,193	55.91 (−2.82)	
SFG, Superior Frontal Gyrus, medial area BA10	7,535	66.45 (−2.78)	
MFG, Middle Frontal Gyrus, area 46	6,299	25.16 (−2.19)	
MFG, Middle Frontal Gyrus, lateral area BA10	6,643	51.78 (−2.76)	
OrG, Orbital Gyrus, orbital area BA12/47	3,726	33.76 (−2.04)	
IFG, Inferior Frontal Gyrus, rostral BA45	2,971		34.26 (−1.90)
OrG, Orbital Gyrus, lateral area BA12/47	4,059	39.10 (−2.10)	
OrG, Orbital Gyrus, lateral area BA12/47	4,714		24.37 (−1.15)
**Temporal lobe**			
STG, Superior Temporal Gyrus, medial area BA38	5,294	33.47 (−2.15)	
STG, Superior Temporal Gyrus, lateral area BA38	2,166	41.92 (−2.31)	
STG, Superior Temporal Gyrus, area 41/42	1,489		49.93 (1.88)
MTG, Middle Temporal Gyrus, rostral area BA21	7,515	33.27 (−2.01)	
**Parietal lobe**			
SPL, Superior Parietal Lobule, rostral area BA7	3,178	32.35 (1.79)	
SPL, Superior Parietal Lobule, intraparietal BA7	3,590	78.38 (1.69)	
IPL, Inferior Parietal Lobule, caudal BA39	9,422	20.83 (1.30)	
IPL, Inferior Parietal Lobule, rostrodorsal BA39	7,928	76.80 (1.73)	
IPL, Inferior Parietal Lobule, rostroventral BA39	10,691	45.49 (1.58)	
POG, Postcentral Gyrus, area BA1/2/3	4,775		39.25 (1.61)
**Insula**			
INS, Insular Gyrus, ventral agranular insula	1,698	34.28 (−2.28)	
INS, Insular Gyrus, hypergranular insula	2,074		34.81 (1.66)
**Cingulate Gyrus**			
CG, Cingulate Gyrus, subgenual BA32	3,250	20.28 (−1.91)	

*Brain areas with a significant activation difference between monaural and binaural listening condition in the N1 time interval in at least 20% of their voxels are listed. Red shading is used for differences in the right hemisphere, while blue shading represents differences in the left hemisphere. Darkest shading indicates a significant difference in at least 75% of the voxels, lightest label is used for differences in less than 25% of the voxels, and tones in between represent categories 50–75% and 25–49% of voxels with a significant monaural vs. binaural difference. Positive t values indicate a larger N1 with bimodal listening, while negative t values indicate a smaller N1 with bimodal hearing. If available, Brodmann areas (BA) are indicated. Note that the spatial extent of differences is larger and pertains to language-associated cortex in CI listeners.*

In NH, activation between listening conditions differed significantly in left primary and secondary auditory cortices (Brodmann areas BA41, 42), i.e., ipsilateral to the side of monaural stimulation (Table 6). The positive *t* value indicated a more negative N1 with binaural listening. In addition, significantly increased negativity in the binaural listening condition was observed in left insula and postcentral gyrus, whereas negativity in the ventral frontal lobe was smaller in the right hemisphere with binaural hearing, indicated by negative *t* values. Affected areas belonged to inferior frontal gyrus (IFG) and orbital gyrus (OrG).

In contrast, differences between electric and bimodal hearing in the CI group affected auditory association areas in the temporal and parietal lobes that are involved in sensory aspects of speech processing (Ardila et al., 2016). Whereas negativity in left temporal areas (BA21, BA38) was smaller, increased negativity was observed in the parietal lobe with bimodal hearing. Affected areas were BA7 and BA39 in the left hemisphere and BA7 in the right. Furthermore, differential activation was observed in the left insula and cingulate gyrus with smaller negativities in the bimodal listening condition.

### Correlations Between AEP and Audiometric Measures

For AEP measures that showed significant differences between listening conditions at T4 and in NH, differences between the binaural minus the monaural condition were calculated and bivariate correlation analyses were performed with these differences and the binaural benefits (Table 4 and section “Binaural Benefits”), and with PTA-4 asymmetry. For the CI group, these were the differences in N1 amplitude related to words, and P2 latency differences in response to words and reversals. For NH, correlations were computed with the difference in N1 amplitude and N1 latency. Only the differences in P2 latencies between monCI and bimodal condition of the CI users showed significant correlations.

The difference in P2 latency in response to words between monaural electric and bimodal hearing was significantly correlated with SU_N_ (*r* = 0.541, *p* < 0.037). A shorter bimodal latency and a larger difference with respect to monaural P2 latency correlated with a larger SU_N_. Furthermore, SU_N_ became negative, which indicates binaural interference, if latency in the bimodal condition was longer than with monaural electric hearing.

In addition, the difference in P2 latency in response to reversals between monaural electric and bimodal hearing was significantly correlated with SU_N_, but here P2 latency in the monaural LC was significantly shorter, and the binaural minus monaural difference in P2 latency showed a significant inverse correlation with SU_N_ (*r* = –0.620, *p* < 0.014), again indicating that a shorter P2 latency in the bimodal listening condition was associated with a larger SU_N_. Thus, in the CI group and for both stimulus categories, a higher SU_N_ was associated with a shorter latency in the bimodal compared to the monaural condition, while a negative SU_N_ can be expected when monaural latency is shorter than latency after bimodal presentation.

## Discussion

Aim of the study was to investigate binaural interactions in bimodal listeners during the early phase of CI use evidenced by AEP and audiometric binaural benefits. With CI experience, the grand-averaged N1 amplitude became increasingly similar to the N1 of NH with an expansion of N1 amplitude in response to words and a reduction of the difference in N1 latency between stimulus categories with bimodal listening. In addition, P2 latency differences between stimulus categories increased for the bimodal condition. Several aspects remained different to NH, however, like the absence of a difference in N1 amplitude between stimulus categories, differences in the localization of brain activity during N1, and the large N2 irrespective of listening condition and stimulus category. The latter has been reported earlier for this group of CI users (Balkenhol et al., 2020). These results indicate that the N1 potential, which is related to the detection of an auditory stimulus, approximates the response seen in NH listeners in some aspects within 6 months of CI provision, including evidence for some binaural integration, albeit at significantly higher presentation levels. Grand average of the later N2 response that has been associated with the effort to understand speech in challenging acoustic situations (Balkenhol et al., 2020) remains different, suggesting continued problems with speech recognition for the bimodal listeners. These findings are in agreement with the CI literature (Sandmann et al., 2015; Finke et al., 2016).

In accordance with AEP results, speech tests at T4 evidenced some binaural integration in the form of a positive SU effect. Together with the increased N1 amplitude in the binaural/bimodal listening condition, this may have been due to an increase in perceived loudness with binaural/bimodal hearing as reported in the literature (Hawkins et al., 1987). Although, bimodal listeners also benefited from the HS effect to a similar extent to the age-matched NH group, this does not indicate central alignment of the electrically and acoustically mediated speech as HS is essentially a monaural effect (van Hoesel, 2012).

A second aim of the study was to explore whether bimodal benefit changes with CI experience. AEP data suggest some improvement in bimodal hearing. It is questionable, however, whether this translates to better speech recognition, in particular in view of the N2 that remains different from NH. Interpretation of audiometric results is more straightforward in this context. At the pre-implantation assessment monaural speech recognition tests were not possible. Whereas this evidences an improvement of speech recognition with CI provision, it prevented estimation of binaural results. At the 3-month interval, performance of the CI ear was worse than performance of the HA ear for a substantial number of the participants. Since adding a better ear in the bimodal condition inflates binaural benefits, these values may rather show a better ear effect. Some degree of binaural benefit was suggested through the significant SU_Q_ effect at the end of the study. While this indicates that the brain can combine the divergent signals transmitted via CI and HA, it requires assessments at a later time to decide whether binaural benefits improve with bimodal experience as suggested by a recent study (Devocht et al., 2017), for instance after performance with the CI ear has reached a stable plateau.

The third aim of the study was to find relevant correlations between binaural benefits and central processing. SU_N_ showed a significant correlation with latency of the P2 potential.

### Binaural Benefits

Study participants continued to use their HA together with the CI, indicating that they accepted this form of hearing provision in their everyday life. Classic binaural benefits are the HS effect based on selection of the ear with better SNR, binaural SU derived from the information being available via two input channels, and the binaural SQ effect which requires central computation of interaural time (ITD) and intensity or level differences (ILD). In addition, speech recognition in noise is improved by spatial separation between signal and noise sources or SRM. Although HS, SU and SQ are largely ascertained for bimodal listeners, not all of them are significant in all published reports (Schafer et al., 2011; Illg et al., 2014; Devocht et al., 2017). Beyond individual capacities and the distribution of hearing ability across ears, the presence and magnitude of binaural effects depends on testing paradigm and material (Schafer et al., 2011), stimulus application (Epstein and Florentine, 2009; Finke et al., 2016), type of masking noise (Illg et al., 2014; Psychny et al., 2014), and the amount of CI experience (Eapen et al., 2009).

The present study group had considerable residual hearing at the HA side, which coincides with participant characteristics from a recent investigation (Devocht et al., 2017), but is distinct to those of earlier reports (Schafer et al., 2011; van Hoesel, 2012). Thus, quantitative comparisons of binaural benefits with those of the earlier reports are possible only to a limited extent. Therefore, bimodal results are mainly compared to the age-matched NH of the current study and to the results by Devocht et al. (2017). As the testing paradigm was similar and participants also used HiRes 90K implants, differences to the current study mainly pertained to longer CI experience (>1 year) and a higher percentage of better CI ears.

The head attenuates sounds at the ear that is shielded from the noise source. Utilization of this HS effect requires the ability to focus on input from the ear with better SNR (Schafer et al., 2011; van Hoesel, 2012). HS was around 4.5 dB SNR, it did not change between T3 and T4, and was not significantly different from the NH group, or experienced bimodal listeners (Devocht et al., 2017). This indicates that our bimodal listeners could exploit HS to a similar extent as NH, and that this does not depend on CI experience, which is consistent with previous findings (Schafer et al., 2011). Results suggest some binaural integration in the bimodal group evidenced by a positive SU. When speech and noise sources coincide in space, the identical signals presented to both ears lead to increased perceptual loudness and improved speech perception. This does not require the listener to use ITD or ILD, but relies on redundancy of the input (Hawkins et al., 1987; Bronkhorst and Plomp, 1990; Endrass et al., 2004; Schafer et al., 2011; Avan et al., 2015). Beyond that, the complementary nature of information transmitted via CI and HA is thought to be an important contributor to SU in bimodal listeners (van Hoesel, 2012). With a T4 group average of 7.7 dB SNR for SU_Q_ and 1.9 dB SNR for SU_N_, SU was similar to that of NH, which in turn was similar to the SU_N_ reported previously for NH using a similar testing paradigm (Bronkhorst and Plomp, 1989). Similarly to the present study, Morera et al. (2005) have reported a reduction of SU between quiet and noise, and a significant SU for the quiet but not for the noise condition. This was for a group of bimodal listeners with about 6 months of CI experience. Furthermore, SU was found to be lower close to threshold (Morera et al., 2005), and in particular when tested at threshold using adaptive paradigms such as the one used in the present study (Schafer et al., 2011). As SU_N_ for the current NH group was as low as in bimodal listeners and SU appears to develop early after CI provision, at least in bilateral CI users (Eapen et al., 2009), the higher SU_N_ of 4.2 ± 0.9 dB SNR reported for experienced bimodal listeners (Devocht et al., 2017) may rather be the result of a difference in sample characteristics rather than more CI experience.

Bilateral symmetrical high-frequency hearing loss has little effect on SU (Hawkins et al., 1987), whereas asymmetry of hearing thresholds reduces SU_Q_ considerably in NH (Heil, 2014). In line with this, symmetry of hearing thresholds, here assessed via CI- and HA-aided PTA-4, correlated significantly with SU_Q_ in the CI group. In accordance, published CI literature suggests that SU is more affected by the interactions between CI and HA performance than by HA performance alone, with greater SU correlating with a smaller difference between CI and HA performance (van Hoesel, 2012; Yoon et al., 2015). In support, a CI simulation study found evidence for a significant binaural integration advantage when the CI simulation ear had a similar level of performance to the other ear (Ma et al., 2016).

In contrast, there was no benefit in spatial unmasking for our bimodal group. Monaural SRM_monCI_ was low and of similar magnitude as for experienced CI listeners (Devocht et al., 2017), while binaural SRM_bin_ was essentially absent (Devocht et al., 2017): 0.8 ± 1.0 dB SNR; current: 1.0 ± 4.0 dB SNR). In contrast, NH listeners of the present study benefited from SRM_monCI_ and SRM_bin_ of about 6 dB SNR each, a finding which is in line with previous work (Bronkhorst and Plomp, 1989). Part of the monaural SRM_monCI_ is attributed to the HS (Williges et al., 2015) and as suggested by the strong and highly significant correlation between these measures (Table 5), while binaural cues that promote SRM_bin_ are ITD and ILD (Papesh et al., 2017). Thus, absence of a binaural SRM effect is interpreted as an inability of the bimodal listeners to exploit ITD and ILD with current technology, due to differences of the temporal and spectral characteristics of sound information transmitted via CI and HA (van Hoesel, 2012).

In agreement with this interpretation, a significant SQ was not evidenced for the bimodal listeners of the current study. Binaural SQ describes the improvement of intelligibility in noise due to addition of input at the contralateral ear with a poorer SNR than in the monaurally active ear (Schleich et al., 2004). It is seen as a binaural phenomenon based on computation of ITD and ILD in the central auditory system (van Hoesel, 2012). With their limited CI experience, our bimodal listeners were not able to exploit ITD and ILD, whereas Devocht et al. (2017) report a SQ of 2.6 dB SNR for experienced bimodal listeners.

Absence of a significant SQ in the OlSa S0NHA condition may have been a result of insensitivity of the adaptive listening paradigm, particularly as a significant SQ could not be shown for the NH group either. A meta-analysis supports this view and suggests that in contrast to supra-threshold testing at fixed SNR levels, the adaptive paradigm may be too insensitive to evidence a SQ, because it is conducted at threshold levels (Schafer et al., 2011). Absence of a significant SQ_AEP_ in the CI group, even at T4, which was tested using a fixed SNR, is in contrast to the highly significant SQ_AEP_ of the NH group, however, and suggests that at this early stage of bimodal experience, there may be no gain derived from SQ. In contrast, the investigation of Morera et al. (2005) on listeners with 6 months of CI experience and testing at a fixed SNR of +10 dB SNR evidenced a significant SQ effect, but here noise was presented at the side of the CI ear. While longitudinal studies investigating the development of the SQ effect in bimodal listeners do not exist as yet, a longitudinal study accompanying bilateral CI recipients over 4 years found SQ to arise at about 12 months after implantation and to continue to increase thereafter (Eapen et al., 2009). Others report significant SQ effects of between 1.9 and 2.9 dB SNR for listeners with more than 12 months of bimodal experience (Kokkinakis and Pak, 2014; Psychny et al., 2014; Devocht et al., 2017; van Loon et al., 2017). One study addressed the effect of adding a contralateral CI in participants with fairly good acoustic hearing (van Loon et al., 2017), and another tested intelligibility in the presence of a speech interferer (Kokkinakis and Pak, 2014) which increases binaural benefits in comparison to noise interferers (Psychny et al., 2014).

Overall, bimodal listeners were able to benefit from HS and SU effects, the latter despite the fact that the input from the two ears was dissimilar, but with limited CI experience of about 6 months they could not benefit from ITD and ILD evidenced by absence of SRM_bin_ and SQ.

### AEP

Together with the studies by Sasaki et al. (2009) and Soshi et al. (2014), this is the only AEP study that addressed bimodal hearing. While Soshi et al. (2014) compared listening in quiet and noise in the bimodal condition and observed reduced N1-P2 amplitudes following speech syllables in noise compared to quiet, Sasaki et al. (2009) compared responses to pure tone stimuli between monaural and binaural listening in a mixed group of bimodal and bilateral CI users and reported shortened latencies of N2 and P3 potentials in the binaural conditions. In contrast, our earlier publication described changes in binaural processing of words with bimodal experience (Balkenhol et al., 2020), and the current investigation explored whether a bimodal benefit develops with CI experience. Bimodal benefit was estimated from differences of N1, P2, and N2 potentials between monaural electric and bimodal hearing in the spatial S0NHA constellation in response to monosyllabic words and their time-reversed sound tracks presented within speech-shaped noise.

NH listeners were expected to show maximal effects and served as a benchmark with which to compare the CI users. Grand average N1, P2, and N2 were present in CI and NH listeners for both listening conditions and in response to both stimulus categories. Several aspects of the N1 and P2 potentials differed between monaural and binaural listening and between stimulus categories in the CI and NH groups, while the late N2 potential differed between groups as reported previously (Balkenhol et al., 2020). As binaural benefits were inflated at T2 and T3 because of the high number of better HA ears, the discussion focusses on the T4 assessment.

A promising result from the present study is the approximation of the difference in N1 amplitude between electric and bimodal hearing to the monaural vs. binaural difference observed for NH listeners. In NH, N1 amplitude in response to words was significantly larger in the binaural condition. In the CI group, the difference in N1 amplitude increased between T2 and T4 and attained statistical significance for the T4 assessment. This finding is in line with the results of a previous study employing an auditory discrimination task to investigate monaural electric hearing (Sandmann et al., 2015), implicating early restoration of N1 amplitudes following CI provision.

Another significant difference between listening conditions pertained to a reduction of P2 latency with binaural listening. While this reduction was observed only in response to words for NH listeners, at T4, shorter P2 latencies in the bimodal condition were observed for both stimulus categories. Significant latency reductions between monaural and bimodal conditions were also reported by Sasaki et al. (2009) following stimulation with pure tones. In that study, latency differences concerned the later event-related N2 and P3 potentials, however. Moreover, Okusa et al. (1999) report longer P2 latencies with increasing task difficulty. Albeit not mentioned explicitly, this finding probably pertained to monaural electric hearing.

The current increase of N1 amplitude and the decrease in P2 latency for binaural/bimodal listening in NH and for the bimodal group at T4 is compatible with an increase in perceived loudness related to binaural loudness summation. The positive SU in NH and at T4 and the significant bivariate correlation between SU_N_ and the P2 latency difference between monaural and bimodal LC at T4 affirm this interpretation. Perceived loudness of a binaurally presented stimulus is louder than its monaural presentation (Hawkins et al., 1987), and there is ample evidence, that N1 and P2 amplitudes increase while their latencies decrease concomitant with the intensity of tones or speech syllables presented in quiet and within background noise (Firszt et al., 2002; Martin et al., 2008; Kim et al., 2012; Sharma et al., 2014; Prakash et al., 2016). Thus, results suggest that with a CI experience of about 6 months, the current sample of bimodal listeners could benefit from SU.

Further factors that influence processing of complex auditory stimuli are familiarity and attention. N1 amplitude was larger while N1 and P2 latencies were reduced in response to word stimuli, although at times pertaining to different comparisons in NH and at T4. Existing literature indicates a stronger response and a more rapid evaluation of familiar stimuli (Kuhl et al., 2007; Kuuluvainen et al., 2014) and suggests that reversed speech sounds are less easily classified within familiar phonetic categories (Binder et al., 2000). Endrass et al. (2004) interpret this as a neurophysiological manifestation of a bilateral redundancy gain, that improves processing of learned meaningful stimuli such as words, but not of complex, unfamiliar, or meaningless stimuli (Endrass et al., 2004). Noteworthy in this context is that significant differences, depending on stimulus categories, were found for more comparisons in NH than in bimodal listeners, suggesting that the familiar sound trace is processed more effectively in NH listeners.

Alternatively, or in addition, attention may have contributed to the difference in N1 amplitudes and P2 latencies related to words and reversals, as suggested previously (Lange, 2013). Our participants were instructed to respond to words but not to reversals. Attention increases amplitude of the N1 for target sounds in CI listeners, but not for distractor sounds (Paredes-Gallardo et al., 2018). Moreover, a study investigating the neural dynamics in the auditory cortex for attending and ignoring showed that responses to the to-be attended stimuli were enhanced around 100 ms post-onset, whereas ignoring led to a decrease in this response (Chait et al., 2010). While a significant difference in N1 amplitude, depending on stimulus category, emerged in NH listeners, current CI listeners did not show such a difference. As CI listeners become more effective at selectively listening to a target stream over time (Paredes-Gallardo et al., 2018), the limited CI experience of the current group may not have been sufficient to produce this effect. Taken together, the most likely interpretation of increased N1 amplitude and shorter P2 latencies in the binaural condition in NH and at T4 appear to be a result of loudness summation. The significant inverse correlation between P2 latency and SU_N_ in CI users affirms this interpretation.

### Source Localization

The grand average of the N1 showed similar monaural vs. binaural amplitude differences in NH and CI groups. N1 consists of several subcomponents with spatially and temporally overlapping neural generators (Näätänen and Picton, 1987), however, leaving the possibility that activation contributing to the N1 response may differ. Based on LORETA source localization, Zhang et al. (2011), suggest spatial and temporal involvement of the following N1 contributors. Upon auditory stimulation, a pre-attentive mechanism in the frontal lobe is activated. If attention is involved, the attention-driven detection of the stimulus is then transmitted to temporal and parietal areas, with involvement of the parietal lobe probably reflecting the matching of sensory information to memory templates. To explore potential similarities and discrepancies between bimodal and NH listeners, source localization analyses were performed for the difference in activation between monaural vs. binaural LCs in the N1 interval. Taking this approach, activity in brain areas that are active to the same extent in monaural and binaural listening does not show, while areas with differential activation are highlighted.

The side of monaural stimulation may influence the N1 response (Gilmore et al., 2009; Hanss et al., 2009) and consequently also the difference between monaural and binaural activation. Therefore, source localization analyses were performed for the subgroups with monaural stimulation of left ears in both groups. Several brain areas exhibited differential activation between listening conditions. Localization differed between CI and NH groups and differences were more widespread in the CI listeners.

For NH, differential activation was found in the left auditory cortex (BA41, 42) with an augmented N1 response in the binaural condition. Organization of the ascending auditory pathways (Malmierca and Hackett, 2009) provides strong evidence for a contra-laterality effect in the N1 interval over the auditory cortex as a function of ear of stimulation and balanced bilateral activation with binaural stimulation (Gilmore et al., 2009). The difference in ipsilateral auditory cortex in NH is therefore interpreted to result from bilateral activation, with binaural presentation leading to a relatively stronger N1 response ipsilateral to the ear that was active in the monaural condition. The increased negativity in left insula in the binaural condition is compatible with the known connections between insula and ipsilateral auditory cortex (Augustine, 1996; Hackett, 2015), and suggests bilateral activation of the insula with binaural listening. The insula is functionally complex and highly connected, and parts of it are seen as a core region of the language system, interfacing sensory and motor language-associated areas (Augustine, 1996; Ardila et al., 2016). Negativity in the binaural condition was reduced in right-hemispheric IFG and OrG, including BA45, which on the left side is regarded as the core of Broca’s area involved in language production (Ardila et al., 2016). Given that increased listening effort has been related to increased activation in the ventral frontal lobe as discussed in Balkenhol et al. (2020), reduced negativity may suggest less listening effort during binaural listening for NH listeners.

The pattern of differential activity in bimodal listeners deviates substantially from that seen in NH. In particular, no difference existed in the primary or secondary auditory cortex (BA41, 42). Possible reasons for this finding are suppression of one ear in the bimodal condition and/or dismantling and reorganization of connections in the auditory system as a result of long-term hearing impairment. It remains to be seen, whether contra-laterality in the auditory pathways increases with continued bimodal hearing.

Differences between electric and bimodal hearing were observed in auditory association areas in the temporal and parietal lobes, mainly in the left hemisphere, and thus in areas that are involved in the sensory aspects of speech processing. Negativity in left temporal areas (BA21, BA38) was smaller with bimodal listening. Left BA21 is part of the core of Wernicke’s area that is involved in sensory aspects of speech processing (Ardila et al., 2016), whereas left BA38 was shown to be sensitive to the acoustic-phonetic contents of human speech (Leaver and Rauschecker, 2010). Reduction of activation in temporal areas in the bimodal condition may suggest less, or less synchronized, activation in these areas with bimodal listening.

On the contrary, N1 negativity increased with bimodal hearing in the parietal lobe, suggesting enhanced processing in parietal areas. Affected areas were BA7 and BA39 (angular gyrus) in the left hemisphere and also BA7 in the right. BA39 is a part of the extended Wernicke area as defined by Ardila et al. (2016) and is thought to be involved in associating language with other types of information (Ardila et al., 2016). Left BA7, in the superior parietal lobe, interacts with regions of the extended Wernicke area, participating in language processing and temporal context recognition (Ardila et al., 2016).

Conversely, during bimodal listening activation was reduced bilaterally in several prefrontal areas including, left BA47, which is part of Broca’s complex and thus involved in language production (Ardila et al., 2016). Activation during bimodal listening was also reduced in the left insula and cingulate gyrus (BA32) that play a coordinating role in interconnecting the perceptive and productive language system (insula), or are associated with cognitive and emotional aspects of language processing (BA32) (Ardila et al., 2016).

Differences between bimodal and NH listeners and the spatial extent of differences in activity, suggest that neuronal circuits differ considerably between groups and between electric and bimodal processing, at least during the initial period of CI use: the latter possibly due to discrepancies in the information conveyed via CI and HA. In general, results from bimodal listeners suggest that contralaterality in the primary auditory cortex is reduced and that a larger part of the cortex involved in language associations is occupied with speech processing during the N1 time window.

### Advantages and Limitations

To the best of our knowledge, this is the first EEG study on bimodal CI users which uses a large set of monosyllabic words. We did this to create a more natural listening situation and to avoid habituation. We could show that this approach is successful in producing several separable AEP. In support of our study design, the present study’s findings are consistent with several other studies investigating speech perception in NH and CI listeners.

Another advantage of our study is that it included an age-matched control group, which allows direct comparison of the amount of binaural benefit that is possible in the testing conditions. A further advantage, but also a potential limitation, is that our CI users used the same CI provision, both in terms of implant and speech processor model being used.

As in other EEG studies with CI users, the major limitation of our study is the small sample size and the heterogeneity of the CI group, which does not permit generalization of the results. Furthermore, advances in CI and HA technology and expansion of implant criteria limit the interpretation of results in relation to former studies, because both personal as well as technical conditions have changed.

EEG data offer high temporal resolution, which is mandatory for describing evolution of the brain’s response to speech stimuli. Because of the inverse problem and the need to employ source localization techniques, there is however, no unambiguous localization of the underlying sources. Therefore, localization data should be interpreted with caution.

Later follow-up would be worthwhile, although this increases the potential problem of worsening of hearing in the HA ear, which has been observed for the current sample and has been noted by others (Sanhueza et al., 2016; van Loon et al., 2017). As significant improvement of monaural electric hearing occurred during the study interval, which obscures the magnitude of benefit derived from binaural input, the study should be replicated with experienced CI listeners, when full employment of the CI ear is expected.

All participants were tested using their own devices with their clinical setting, because the purpose of the study was to evaluate the binaural benefits in everyday use, as opposed to the effect of optimal and synchronized CI and HA fitting. Results imply however, that to gain maximal binaural benefit, amplification in one or the other device may have to be reduced, as suggested by a reduction of SU with asymmetry of the PTA-4.

## Conclusion

Major findings of the study are the following:

• With 6 months of CI experience, bimodal listeners were able to make use of the HS and SU effects but did not benefit from SQ or SRM, indicating insufficient alignment of electrically and acoustically transmitted auditory signals in the central auditory system.
• The significant correlation of binaural SU with the bimodal/monaural CI latency difference of the AEP response confirms its potential use as an objective measure for the quality of bimodal hearing.
• EEG results for the bimodal group demonstrated N1 responses that were similar to NH listeners in terms of magnitude and response characteristics.
• Source localization reveals distinct processing for bimodal listeners in the N1 interval, however, suggesting loss of lateralization in the auditory system and augmented associative processing in speech relevant areas. Therefore, it will not be sufficient to use an averaged N1 response to estimate the quality of bimodal processing.

## Data Availability Statement

The datasets presented in this article are not readily available because of ethical or legal reasons. Requests to access the datasets should be directed to EW-F, elisabeth.wallhaeusser-franke@medma.uni-heidelberg.de or TB, tobias.balkenhol@medma.uni-heidelberg.de.

## Ethics Statement

The studies involving human participants were reviewed and approved by Institutional Review Board of the Medical Faculty of Mannheim at Heidelberg University. The patients/participants provided their written informed consent to participate in this study.

## Author Contributions

TB designed the computational framework, collected and analyzed the data, and wrote the manuscript. EW-F designed the study, collected and analyzed the data, and wrote the manuscript. NR did critical review. JS recruitment, collection of clinical data, and critical review. All authors contributed to the article and approved the submitted version.

## Conflict of Interest

The authors declare that this study was partly funded by Advanced Bionics, Staefa, Switzerland. Advanced Bionics AG manufactures the device under investigation in this study. The funder was not involved in the study design, collection, analysis, interpretation of data, the writing of this article or the decision to submit it for publication.

## Footnotes

1 http://www.playrec.co.uk

2 http://www.ru.nl/fcdonders/fieldtrip
